# Key effects of inclusive dance for adults with developmental disabilities: embodied agency, relational self-understanding, and conditions of wellbeing

**DOI:** 10.3389/fpubh.2026.1934739

**Published:** 2026-09-11

**Authors:** Hyunkyun Ahn, Dongyoon Shin, Deok-Jin Jang

**Affiliations:** 1 Department of Sport and Leisure Studies, Division of Arts and Health Care, Myongji College, Seoul, Republic of Korea; 2 Graduate School of Education, Physical Education, Sogang University, Seoul, Republic of Korea; 3 Department of Sports Medicine, Shinhan University, Uijeongbu-si, Republic of Korea

**Keywords:** critical phenomenology, developmental disabilities, embodied agency, inclusive dance, normative visibility, qualitative research

## Abstract

**Introduction:**

This study explored the inclusive dance experiences of adults with developmental disabilities through the lens of embodied agency and critical phenomenology. Most previous research on arts participation has focused on outcomes such as emotional stability, social connectedness, and quality of life. However, less is known about how arts participation, specifically inclusive dance, is experienced and sustained. This study examined how rehearsal and performance experiences engender embodied possibilities, relational self-understanding, and conditions of wellbeing.

**Methods:**

This qualitative case study included three adults with developmental disabilities who had accumulated between 9 and 13 years of participation in an inclusive dance company in Seoul, along with their primary caregivers. Data were collected through in-depth interviews and observations, focusing on repeated rehearsals and performance practices, everyday experiences, and relational interactions. The data were analyzed through repeated readings to identify key themes, using a phenomenological perspective for directional interpretation.

**Results:**

Inclusive dance participation was experienced as a process through which participants encountered their bodies and possibilities anew through repeated performances, relational coordination, public recognition, and environmental flexibility. Rehearsal and performance enabled participants to experience their bodies as capable, while their sense of agency was shaped by moving with others and adjusting to shared performance situations. Their performance experiences extended their self-expression and relational self-understanding through public recognition and role-taking. Sustained participation was maintained and negotiated through bodily conditions, temporal arrangements, mobility support, caregiving relationships, and flexible performance environments.

**Discussion:**

This study interprets inclusive dance as a participatory experience in which the body, relationships, norms, and everyday world intersect for adults with developmental disabilities. Its contribution lies in suggesting that the meaning of arts participation cannot be understood only as a psychological change or program effect. Rather, inclusive dance can be understood as a process involving embodied agency, normative visibility, relational self-understanding, and conditions of sustained participation.

## 1 Introduction

Arts engagement is closely entwined with our sense of social connection, meaning-making, recognition, and identity. Research on such engagement with the arts and its impact on health has moved beyond documenting the benefits of artistic engagement to examining the experiences and social processes through which arts participation supports wellbeing. Thus, it is important to gain a better understanding of how these processes can develop within certain relational and environmental conditions to enhance their benefits (1).

Some studies show that arts engagement in adulthood can support daily social connectedness (2); for example, arts and cultural participation during the COVID-19 pandemic provided relational resources and reduced isolation (3). Research on adolescents and young people also suggests that regular participation in the arts and creative activities improves subjective and relational wellbeing (4). These studies have examined the relationship between arts engagement and wellbeing by considering the relational, interpretive, and cognitive processes through which arts participation becomes meaningful.

Arts participation may also contribute to health through forms of embodied activity that connect movement, expression, social interaction, and emotional experience. Dance is especially relevant in this regard because it is simultaneously an artistic practice and a form of physical activity. It combines bodily movement and coordination with creative expression, social connection, rehearsal, and public participation. Its relationship with health therefore cannot be reduced either to physical fitness outcomes or to artistic experience alone. Rather, dance may contribute to health and wellbeing through the interaction of bodily, relational, emotional, and social processes.

Research on arts-based interventions in health and wellbeing contexts nevertheless faces conceptual and methodological challenges. The heterogeneity of intervention components and outcome measures, together with difficulties in establishing appropriate comparison groups, can limit the interpretation of findings (5). Similar concerns have been raised in studies of group-based performing arts interventions, in which program design, participation contexts, and evaluation indicators vary across studies (6). These limitations are particularly important when health is treated primarily as an outcome to be measured, because such an approach may provide limited insight into how participants experience bodily change, how that change is recognized by others, and under what conditions participation becomes meaningful and sustainable. Research therefore needs to address not only whether arts participation is associated with health-related outcomes, but also how it's embodied and relational processes are experienced within particular social settings.

This issue is particularly important in research on disability arts. Arts inclusion cannot be adequately established through program access or short-term outcomes alone. Inclusion concerns the right to participate in cultural life and should be examined through the concrete conditions in which accessibility, social stereotypes, role allocation, and institutional support operate together (7, 8). Among people with disabilities, cultural participation may be constrained by institutional barriers, limited accessibility, negative attitudes, and insufficient involvement in decision-making within cultural organizations (9). Thus, the social meaning of disability arts will not only be determined by individual inclusion but also by how participation is interpreted and sustained within relational networks, institutional contexts, and cultural settings (10). For adults with developmental disabilities, these questions have particular significance. Adults with developmental disabilities may have diverse cognitive, communicative, adaptive, and social support needs (11). Their participation, however, is also shaped by environments that privilege particular forms of bodily competence, communication, independence, and public presentation (12, 13). Conventional dance settings may therefore become exclusionary when established standards of technique, learning pace, bodily control, or performance are treated as universal requirements. Exclusion in this context does not arise solely from an individual's impairment. It is also produced through normative assumptions about which bodies can dance, how dancers should learn and perform, and whose bodily expression is considered appropriate for public visibility (12, 14).

Inclusive dance has emerged as an approach that broadens opportunities for participation by accommodating diverse bodily capacities and forms of expression within a shared artistic and movement practice (15). Rather than requiring all participants to conform to a predetermined bodily norm, inclusive dance may reorganize instruction, rehearsal, choreography, communication, and role allocation around different forms of participation. Its significance therefore lies not simply in allowing entry into an existing dance setting, but in reconsidering how dancing, performing, and participation are organized. It may consequently provide adults with developmental disabilities with opportunities for physical movement, creative expression, relational coordination, and public participation, while also involving the expectations and responsibilities associated with rehearsal and performance.

Previous studies report that dance and expressive arts participation are associated with autonomy, self-expression, social behavior, and emotional experience (16, 17). Research on dance involving adults with intellectual and developmental disabilities has also focused on physical functioning, social interaction, and self-expression (18). Other studies have explored the possible relationship between virtual exercise or dance programs and fitness or functional ability (19). This body of research indicates that dance may be relevant to physical, emotional, and social dimensions of health. Much of the existing literature; however, has assessed participation through functional improvement, behavioral change, or measurable health effects. Such findings provide less insight into how participants experience changes in bodily possibility, how recognition and visibility shape these experiences, and whether they extend beyond the program into everyday life. The relationship between inclusive dance and health therefore requires a broader qualitative examination that considers how participants experience bodily possibility, social recognition, and participation within everyday life. Rather than treating health and wellbeing solely as measurable outcomes, this study examines how participants experience themselves as capable through embodied action, relational recognition, and meaningful participation. A qualitative approach is therefore appropriate for examining how bodily movement becomes connected to agency, public recognition, and everyday self-understanding.

Several questions consequently remain insufficiently addressed. How is change experienced by adults with developmental disabilities participating in inclusive dance? Under what relational and normative conditions is bodily possibility recognized or constrained? How do rehearsal and public performance shape participants' social visibility? Finally, how do these experiences extend into everyday self-evaluation, self-expression, relationships, and wellbeing? Addressing these questions requires an approach that examines inclusive dance as a lived and relational practice in which bodily possibility, social recognition, public visibility, and health are configured together.

This study examined the experiences of adults with developmental disabilities participating in an inclusive dance program, together with the accounts of their caregivers. Particular attention was given to rehearsals and public performances as settings in which bodily action, relational coordination, and public visibility became intertwined. The analysis focused on how participants experienced bodily possibilities, how these possibilities were recognized through feedback and role allocation, and how performance experiences became connected to everyday conditions of wellbeing.

Dance is particularly relevant for examining embodied agency because bodily possibilities emerge through movement, rehearsal, and relational coordination. Drawing on Merleau-Ponty's notion of the “I can,” embodied agency may be understood as the practical experience of discovering what the body can do within particular situations and relationships (20). Rehearsals and performances, therefore, provide experiential settings in which participants encounter new possibilities for action and self-understanding (21–23). These experiences also unfold within broader social and relational conditions in which recognition, normative expectations, and visibility shape participation (12, 14, 24, 25). Together, phenomenology and critical phenomenology provide the conceptual basis for examining embodied agency, normative visibility, and wellbeing as interconnected dimensions of inclusive dance participation.

Accordingly, the objective of this study was to examine how embodied agency and normative visibility experienced through rehearsal and public performance become connected to the everyday conditions of wellbeing of adults with developmental disabilities participating in inclusive dance. The study was guided by the following questions. First, how is embodied agency, particularly the sense of the “I can,” experienced and developed in inclusive dance participation among adults with developmental disabilities? Second, under what conditions do naming, feedback, and audience responses in rehearsal and public performance shape participants' normative visibility and experiences of wellbeing? Third, how do experiences of recognition and visibility in public performance extend into everyday self-evaluation, self-expression, and relational practice, and how are pride and burden organized together in this process?

## 2 Theoretical framework

### 2.1 Embodied agency and the phenomenology of the “I can”

Inclusive dance participation among adults with developmental disabilities can be understood as a process in which the body's relationship with the world is reorganized. Merleau-Ponty's (20) phenomenology of the body provides a philosophical basis for this, conceptualizing the body as a subject that perceives, moves, and generates meaning in relation to the world. In the present study, the concept of the “I can” serves as the primary interpretive lens for understanding how participants experience and expand embodied possibilities through inclusive dance participation. In inclusive dance, the “I can” concerns how participants experience what their bodies can do within unfamiliar spaces, in relation to the movements of others, repeated rehearsal, and the demands and uncertainties of performance. This concern with what a body can do also connects with disability-studies scholarship that, through a feminist materialist lens, rethinks bodily capability apart from deficit or appearance and situates it in the bodies' material and relational engagements with the world (13). Recent studies in disability phenomenology and critical phenomenology also suggest that such possibilities are not fixed attributes but are continually adjusted through relational support and environmental conditions (26, 27). These perspectives extend phenomenological accounts of embodiment by emphasizing that agency emerges through situated relationships and environmental engagement rather than through individual capacity alone (26, 27).

Body schema and habit have been widely discussed within phenomenological and embodied approaches to agency (20). Body schema refers to how the body positions and adjusts itself in relation to space, others, and objects. Habit explains how repeated movements stabilize the body and create new possibilities for action. In this study, body schema and habit are understood as complementary processes through which the sense of the “I can” gradually develops. Whereas, body schema enables the body to orient itself within changing situations, habit explains how repeated engagement with movement expands the body's practical field of action over time. In dance, repeated rehearsal is more than memorizing movements. It involves coordination, rhythm, direction, distance, and intersection with the movements of others. Through repeated rehearsal, participants develop new bodily habits that progressively reorganize their body schema, allowing previously unfamiliar movements to become familiar possibilities for action. Contemporary phenomenological approaches similarly suggest that bodily habits are continually reorganized through ongoing participation in social, relational, and environmental contexts, allowing new possibilities for action to emerge over time (26, 27).

Expressive meaning is another important dimension of Merleau-Ponty's (20) phenomenology. Bodily movement is a mode of expression through which the body discloses its relationship with the world. The body expresses emotions, relationships, and self-understanding in ways that precede language, with these expressions taking shape through movement. In dance, movement is a means by which participants perceive their bodies differently and develop new forms of self-understanding. Purser (22) described dance as a way of generating knowledge through movement. This perspective provides a theoretical basis for analyzing how inclusive dance participation may reshape self-understanding and relational experiences.

Phenomenological and disability phenomenological perspectives emphasize that bodily experience is formed in relation to others and the environments in which action becomes possible (20, 26, 27). Thus, bodily experiences are not formed by individuals in isolation. They are modulated and affirmed through intercorporeality, that is, the reciprocal and embodied coordination through which bodies respond to and orient themselves toward one another (20). Contemporary disability phenomenology further highlights that interdependence is not a limitation on agency but a condition through which agency is enacted and sustained (26, 27). In rehearsals and performances, participants' movements are shaped by others who move with them, by feedback, and by the rhythm and rules of the scene. Purser (21) explained that dance experiences develop through coordination with others and shared movements. From this perspective, agency in inclusive dance is not the fixed capacity of an individual but a relational possibility that is stabilized through movement and coordination with others. Within this framework, the sense of the “I can” is gradually formed as participants negotiate bodily and environmental conditions through repeated performance, relational support, and coordination with others (26, 27).

Taken together, phenomenological and disability phenomenological perspectives, supported by contemporary embodied approaches, provide the conceptual framework for interpreting inclusive dance participation as a process of embodied agency (20, 26, 27). Within this framework, body schema and habit account for the reorganization of bodily possibilities through repeated practice; expressive meaning illuminates how movement shapes self-understanding; and intercorporeality clarifies how agency develops and is sustained through relations with others. Together, these concepts provide a coherent basis for interpreting how bodily, relational, and environmental experiences contribute to the gradual development of the sense of the “I can.”

### 2.2 Normative visibility and conditions of participation

The embodied possibilities developed through inclusive dance are experienced within specific social, spatial, and relational conditions. The sense of being able to move is not merely an internal bodily experience. It also relates to how particular bodies are seen as capable of performing in public settings. Critical phenomenology is useful because it does not reduce experience to an individual psychological state. Instead, it examines the normative, spatial, political, and relational conditions that make particular experiences possible (12). From this perspective, inclusive dance can be understood as a process through which bodily possibilities are made visible, recognized, and negotiated within public and relational settings. In this study, normative visibility refers to the socially organized conditions through which particular bodies and movements become recognizable as appropriate, capable, or meaningful in public performance. Rehearsals and performances are public settings in which participants encounter how their bodies and movements are seen, evaluated, and recognized by others. Visibility in inclusive dance is therefore not simply a matter of being seen but of whether particular bodies and movements become socially recognizable as meaningful forms of performance. Such visibility may affirm pride and social presence while simultaneously exposing participants to evaluation and normative expectations.

Studies in critical phenomenology challenge the assumption that bodily experience is shaped in socially neutral environments. Weiss et al. (12) argued that the designation of particular bodies as “normal,” “appropriate,” or “fitting” is inseparable from the social norms through which bodies are perceived and evaluated. In inclusive performing arts, performing bodies are continually interpreted through role expectations, performance structures, feedback, and audience responses. Participation therefore concerns not only access but also how bodies are arranged, how performance norms are organized, and under what conditions recognition becomes possible. Garland-Thomson's concept of “misfitting” (14) offers an important analytic language for interpreting these experiences. The conditions of participation thus extend beyond physical accessibility to include the normative and relational organization of rules, feedback, role allocation, and the interpretation of mistakes and failure (28).

The right to cultural participation places these relational and normative conditions within a broader institutional context. The UN Convention on the Rights of Persons with Disabilities identifies participation in cultural life as a fundamental right (8). Recent studies have examined how cultural participation is restricted by institutional barriers and social stereotypes (29). Thus, inclusive dance is more than a shared activity. It is a cultural setting in which meaningful participation depends on whether disabled people are afforded substantive roles and recognized as performers rather than merely included as attendees.

Ahmed's concept of orientation (24) further clarifies how normative visibility is organized spatially. Orientation concerns how bodies are directed through spaces, objects, and established pathways, and how some forms of movement become familiar and readily accommodated while others are rendered difficult or out of place. Applied to inclusive dance, this concept draws attention to how rehearsal spaces, movement sequences, performance positions, and expected ways of moving may facilitate some bodies while constraining others. This perspective shifts attention away from narratives of individual overcoming and toward the normative and spatial arrangements through which bodily possibilities are enabled or restricted.

Recognition in public performance can also shape participants' self-evaluation and self-understanding. Being recognized as a performer may affirm bodily capability, strengthen social presence, and contribute to more positive forms of self-understanding. At the same time, recognition may introduce new performance expectations and responsibilities. Recent studies in disability embodiment emphasize that recognition can create a sense of possibility while generating new standards of performance and demands for responsibility (30, 31). Recognition is therefore not an unconditionally positive outcome but a relational experience in which affirmation, vulnerability, and responsibility may coexist.

From a critical phenomenological perspective, inclusive dance participation is an experience in which bodily possibility unfolds while being adjusted through norms and relationships. Normative visibility clarifies how particular bodies and movements become publicly recognizable as capable and meaningful; misfitting shows how participation depends on the alignment between bodies and performance conditions; and orientation explains how spatial and social arrangements direct the possibilities available to different bodies. Together, these concepts frame inclusive dance as a relationally, spatially, and normatively organized process through which embodied possibility, self-experience, and wellbeing emerge in public participation rather than within the individual alone.

### 2.3 Integrating phenomenology and critical phenomenology as an interpretive framework

Merleau-Ponty's (20) phenomenology, together with disability phenomenological and critical phenomenological perspectives, illuminates inclusive dance participation at different but connected levels. Merleau-Ponty's phenomenology of the body explains how the body develops possibilities through its relation to the world. Critical phenomenology examines how embodied experiences become possible within social norms, spatial arrangements, and relational settings (12). Their integration enables inclusive dance participation to be interpreted as a situated process in which embodied possibilities develop through, rather than independently of, the normative and relational conditions of participation.

Embodied agency concerns the process through which the body develops a sense of the “I can” in relation to its environment. Such agency, however, is always situated: participants' movements acquire meaning through spatial arrangements, interactions with others, role expectations, feedback, and the conditions of public performance. Phenomenology clarifies how participants perceive, enact, and expand bodily possibilities, while critical phenomenology examines how those possibilities are enabled, constrained, and recognized within particular social and spatial arrangements. These perspectives are therefore not applied as separate explanatory layers. Instead, they converge in rehearsals and performances, where participants' bodily possibilities are simultaneously developed, negotiated, and made publicly meaningful.

This framework also helps avoid separating inclusive dance participation into isolated dimensions, such as function, emotion, or social participation. Embodied possibilities do not arise solely within the individual body, just as normative conditions do not operate merely as external constraints. Rather, bodily experience and participation conditions are mutually constituted through movement, interaction, and public recognition. Participants' sense what their bodies can do while experiencing how such possibilities are perceived and recognized by others. The analysis therefore focuses on the relational processes through which inclusive dance becomes both an embodied practice and a publicly recognized form of participation.

This integration is also central to the study's understanding of wellbeing. Wellbeing is not treated solely as emotional stability, positive affect, or an individual psychological outcome. It is understood as a situated experience that develops when participants encounter their bodies as capable of meaningful action and are able to sustain that possibility through recognition, relational support, and enabling participation conditions. From this perspective, inclusive dance may contribute to wellbeing not simply because it produces personal change, but because it creates conditions through which embodied agency and socially meaningful participation can be developed and maintained.

In sum, Merleau-Ponty's phenomenology provides the basis for interpreting how bodily possibilities are experienced and developed, while critical phenomenology clarifies how those possibilities are shaped by normative, spatial, and relational conditions. Together, these perspectives position the dance experiences of adults with developmental disabilities as a mutually constitutive process in which embodied agency, normative visibility, and the conditions of participation continually shape one another.

## 3 Methodology

### 3.1 Study design

To ensure a lived and relational setting for our investigation, our study was designed as a qualitative inquiry informed by hermeneutic phenomenology to interpret the inclusive dance experiences of adults with developmental disabilities (32, 33). We focused on how participants experienced and interpreted their bodies, roles, and relationships with others in rehearsals and public performances. Hermeneutic phenomenology is appropriate for interpreting the meaning and structure of lived experiences as they are formed within the real world (32). Accordingly, we approached inclusive dance participation as an experiential process in which bodily possibility, relational coordination, and public visibility are interrelated. Whereas, hermeneutic phenomenology guided the interpretation of participants' lived experiences and the meanings they attributed to them, critical phenomenology provided a complementary perspective for understanding how those experiences were shaped by broader relational, normative, and environmental conditions.

The study environment was an inclusive dance company located in Seoul, Republic of Korea. The company provided an ongoing inclusive dance program for adults with developmental disabilities, in which regular practice sessions, rehearsals, performance preparation, and public performances formed the core structure of participation. These settings constituted the primary contexts for the present study. This allowed us to interpret participants' experiences within the concrete settings in which they occurred and to examine the experiential structure of inclusive dance participation through specific rehearsal and performance situations. The inclusive dance company was understood as the experiential setting in which participants' embodied agency, role experiences, and conditions of recognition occurred. The study was grounded in an interpretivist epistemology. Experience was not treated as a direct representation of fixed facts but as a process through which participants understood and interpreted their bodies and actions within particular situations and relationships (34). From this perspective, interview accounts and observation notes were used as complementary data sources for understanding how the participants' experiences were expressed through verbal accounts and situational contexts. Critical phenomenology informed the interpretation of how these experiences were shaped by relational, normative, and environmental conditions, including role allocation, feedback, social recognition, and public visibility. This approach enabled us to interpret both the meanings participants attributed to their experiences and the relational conditions through which those meanings emerged.

### 3.2 Participants and recruitment

We interviewed three adults with developmental disabilities who had participated continuously for more than a year in the inclusive dance company, along with their three primary caregivers. As our aim was to understand the experiential structure of inclusive dance participation and the formation of everyday meaning, purposive sampling was used to recruit these participants with accumulated rehearsal and performance experience (35). Participants were eligible if they had participated regularly in the dance company for at least 1 year, were able to participate in interviews, and could describe their rehearsal and performance experiences verbally. These criteria were intended to ensure that participants could reflect on concrete experiences accumulated through sustained participation in the dance program. Accordingly, all participants were adults with developmental disabilities who were able to communicate verbally during interviews and had accumulated extensive rehearsal and public performance experience. Their continued participation in rehearsals and public performances enabled them to reflect on embodied, relational, and performance experiences grounded in specific situations within the dance program. Interviews with caregivers were used as supplementary data to understand participants' accounts within their everyday relational contexts (34). Caregiver accounts were treated as contextual and complementary sources for interpreting participants' everyday experiences rather than as substitutes for participants' own accounts. In particular, caregiver data helped clarify how dance participation was interpreted in relation to daily life, relational experiences, and role perceptions. Recruitment was conducted in cooperation with the dance company, which provided information about the study to eligible participants and their caregivers. Decisions regarding participation and consent were made through direct communication with the authors. To reduce the potential influence of organizational involvement, the dance company only provided initial information about the study and facilitated contact with eligible participants. Consent was obtained directly by the research team after participants and caregivers had received an explanation of the study and confirmed their willingness to participate (36). Participant characteristics are summarized in Table 1.

**Table 1:** Participant characteristics.

Participant	Gender	Age	Disability type	Communication	Role	Years of participation
Doyoon	Male	30s	Developmental disability	Verbal; independent expression	Dancer	13 years
Yujin	Female	20s	Developmental disability	Verbal; occasional interviewer prompting	Dancer	12 years
Jia	Female	20s	Developmental disability	Verbal; occasional clarification	Dancer	9 years
Caregiver of Doyoon	–	–			Contextual informant	
Caregiver of Yujin	–	–			Contextual informant	
Caregiver of Jia	–	–			Contextual informant	

### 3.3 Sample size and data collection

The sample size was determined based on the analytical purpose of the phenomenological inquiry and the bounded nature of the case. In phenomenological research, sample adequacy is not determined solely by the number of participants. It also depends on the depth of experience relevant to the research purpose and the interpretive potential of the data (37). We examined the meaning structure of inclusive dance participation through repeated interviews, caregiver interviews, and non-participant observations. In addition, all three participants had continuously experienced similar rehearsal and performance structures within the same dance company, supporting contextual consistency and comparability across their accounts. Taken together, these characteristics provided sufficient experiential depth and contextual consistency to support an in-depth phenomenological interpretation of inclusive dance participation (37, 38).

Data were collected between October and December 2025 through in-depth interviews and non-participant observations. The interviews were semi-structured and designed to help participants recall and describe their dance experiences in relation to specific scenes (39). The interview questions explored participants' bodily experiences, interactions with others, experiences of receiving feedback from others, role-taking during rehearsals and performances, and changes in everyday self-understanding. During the interviews with the participants with developmental disabilities, we presented the questions as short, concrete units. When the meaning of a response was unclear, we checked for understanding by summarizing the participants' expressions and asking brief follow-up questions (40). The interview duration was flexibly adjusted to accommodate participants' fatigue and changes in concentration. Individual interviews with participants with developmental disabilities lasted approximately 40–70 min, whereas caregiver interviews lasted approximately 60–90 min. When necessary, interviews were divided into more than one session. Abstract questions were minimized, and participants were encouraged to recall their experiences through concrete scenes, such as the rehearsal room, stage, feedback, and audience responses. In total, we conducted 12 interviews: three with each of the three participants with developmental disabilities and one with each of the three caregivers. All interviews were conducted in Korean, audio-recorded with participants' consent, and transcribed verbatim. Quotations presented in this manuscript were translated into English by the authors. To maintain the meaning of participants' accounts, translated quotations were repeatedly checked against the original Korean transcripts during the analysis and manuscript preparation process. Interviews with participants with developmental disabilities were conducted in settings in which they felt comfortable and secure. Caregiver interviews focused on understanding the participants' everyday contexts and relational changes following dance participation.

We supplemented the interviews with non-participant observations of the main activities of the dance company. These observations covered regular practice sessions, rehearsals, performance preparation, and performances. Each observation lasted for approximately 2–3 h and 10 observation sessions were conducted in total. Our observations focused on how bodily coordination, interaction, role allocation, and feedback were organized in rehearsals and performances. The participants' movements, interactions with peers and instructors, role performance, and the emotional atmosphere and flow of each scene were recorded in field notes (41, 42). These notes were reviewed repeatedly alongside the interview data. The observation data provided contextual evidence for interpreting participants' accounts and understanding the rehearsal and performance situations in which those experiences were embedded (43). The integration of participant interviews, caregiver interviews, and observational field notes enabled comparison across multiple sources of evidence while preserving participants' own accounts as the primary basis for interpretation. This approach supported an in-depth interpretation of participants' lived experiences and the relational conditions through which those experiences were shaped.

### 3.4 Data analysis

Data analysis followed hermeneutic phenomenological procedures for thematic interpretation (32, 33). Our analysis focused on interpreting the experiences and relationships in inclusive dance participation. We repeatedly read the interview transcripts, caregiver interview transcripts, and observational field notes to examine how the participants experienced and made sense of their bodies, roles, relationships with others, and public visibility in the rehearsal and performance settings. In the first stage of analysis, these materials were examined to develop an overall understanding of the dataset. Meaning units were identified from specific scenes, expressions, emotional responses, and relational interactions that captured the participants' experiences. The initial meaning units were first coded descriptively and then compared across participant interviews, caregiver interviews, and observational field notes before being grouped into provisional themes. These units were developed by considering the content of individual accounts, the context of each scene, and their everyday-world meanings. We sought to preserve participants' expressions as much as possible while examining the relationships and conditions through which meanings were formed. The analysis proceeded through a hermeneutic cycle. This means that we moved repeatedly between interview accounts and observed scenes and between the individual accounts and the entire dataset to refine interpretation. The initial meaning units were grouped into provisional themes, which were revised and adjusted through repeated rereading of the data. The first author conducted the initial analysis and developed the preliminary thematic structure. These preliminary interpretations were subsequently reviewed and discussed among all authors, who examined the coherence of the themes, their fit with the data, and the boundaries between themes. Through this collaborative process, the thematic structure was refined and finalized. The theme boundaries were established by considering recurrence within the data, connections across scenes, and similarities and differences among the participants.

After the preliminary thematic structure had been developed, critical phenomenology was used as the theoretical perspective for interpreting the relational, normative, and environmental conditions through which participants' experiences became possible (12). We examined how the participants' experiences were shaped and given meaning within particular scenes and relationships. This perspective informed the interpretation of bodily possibilities, role allocation, feedback, gaze, and boundaries of participation. Caregiver interviews and observational data were used to interpret the situated and everyday contexts of the participants' accounts. Caregiver data helped clarify how dance participation was connected to everyday self-understanding, relational experiences, and perceptions of roles. Observational data were used to examine how the experiences mentioned in the interviews appeared in actual interactions during rehearsals and performances. For example, a participant's interview account of continuing to dance through moments of difficulty was complemented by an observed episode in which she returned to rehearsal after temporarily stopping because of a calf cramp. The observation showed how continued participation was enacted in the rehearsal setting rather than only described retrospectively in interview. In another instance, a participant's account of being addressed as a “teacher” and experiencing recognition through this role was complemented by his caregiver's report that positive evaluation led him to try harder and develop a greater sense of responsibility. These sources were compared during theme development to examine how participant accounts were confirmed, refined, or extended by caregiver accounts and observations. Throughout the analytical process, analytic memos were maintained to document the basis for theme development, changes in interpretation, and reasons for adjusting the boundaries between themes. Inconsistent or exceptional scenes were used to reconsider the scope and meaning of themes. Through these procedures, the final themes were refined into coherent interpretive units grounded in participants' accounts and supported across participant interviews, caregiver interviews, and observational data.

### 3.5 Methodological rigor

Methodological rigor was guided by Lincoln and Guba's criteria of trustworthiness (credibility, confirmability, transferability, and dependability) (44, 45). Credibility was strengthened through the combined use of repeated interviews, caregiver interviews, and observational field notes (46). Participants' accounts were compared with caregiver interviews and observational data throughout the analytical process to examine whether similar experiences and meanings were evident across different contexts and sources. Confirmability was supported by documenting the analytic links among the original data, meaning units, and themes, allowing the analytic process to be traced from the original data through successive stages of interpretation to the final themes. These procedures enhanced the traceability of the interpretation and helped examine the connections between the data and analytic claims. Transferability was addressed by providing detailed descriptions of the research context and participation setting. As this study was conducted within the bounded context of one inclusive dance company in Seoul, Republic of Korea, emphasis was placed on contextual understanding rather than broad generalization. Instead, we sought to provide sufficient contextual detail to demonstrate how participation experiences were shaped by particular conditions and relationships. To that end, the activity structure of the dance company, rehearsal and performance settings, participant composition, and observation contexts were described in detail. Dependability was supported by consistent documentation of the main data collection and analysis procedures. Key analytic decisions made during the interviews, transcription, observation recording, meaning unit development, coding, and theme refinement were systematically documented throughout the research process. Comparisons across data sources and revisions to the thematic structure were repeatedly reviewed through discussions among all authors to enhance the consistency of interpretation. The research team brought complementary academic and practice-based perspectives to the study. Collectively, the authors have experience in disability sport research, disability-related practice, dance, and inclusive arts education. These complementary backgrounds informed the study's attention to embodied experience, participation conditions, and social recognition, while also creating the possibility that prior understandings of disability, inclusion, performance, and arts participation could influence the research design and interpretation of the data. Reflexivity was therefore maintained through ongoing collaborative discussions, in which the authors critically examined their assumptions, considered alternative interpretations, and sought to keep participants‘ accounts central throughout the analytical process (47).

### 3.6 Ethical considerations

This study was reviewed and approved by the Institutional Review Board of Sogang University (IRB No. SGUIRB-A-2510-67). We explained the purpose of the study, data collection procedures, anonymity, data storage, and the right to withdraw to all participants and caregivers. Written informed consent was obtained from the caregivers, and the participants' assent was confirmed before data collection. The study was explained to participants with developmental disabilities using accessible language and concrete examples. During the interviews and observations, we monitored the participants' willingness to continue and checked for signs of fatigue or discomfort (36). Interviews were paused, stopped, or rescheduled when the participants experienced fatigue or discomfort. All data were de-identified and stored in encrypted files. Access to the data was restricted to the authors. When reporting the findings, personal information and contextual details were modified as necessary to prevent identification of individual participants. Potential identifying information, including age, place of residence, and occupation, was reported in categorized form to protect confidentiality.

## 4 Results

We identified three interrelated themes: the formation of embodied agency, normative visibility and participation conditions, and the everyday continuation of performance experience. These themes were developed to reflect recurring patterns across the participant interviews, caregiver interviews, and observational data and to capture the experiential process through which participants developed a sense of embodied possibility within dance practice, encountered recognition and normative expectations in performance settings, and carried these experiences into everyday life. Together, the themes illustrate how inclusive dance participation was experienced not as a single event but as a process linking embodied action, public recognition, and the ongoing conditions of participation. Each theme was supported by evidence drawn from multiple data sources, including participant interviews, caregiver interviews, and observational records; Table 2 presents the final themes, subthemes, and supporting data sources.

**Table 2:** Final themes, subthemes, and supporting data sources.

Final themes	Subthemes	Supporting data sources
Embodied agency formation	Repeated performance and the reconstruction of possibility	Participant interviews, caregiver interviews, and observational records
	Feedback and the embodiment of performance rules	Participant interviews, observational records
	Relational coordination and the confirmation of agency	Participant interviews, observational records
Normative visibility and conditions of participation	Legitimizing participation through naming and role allocation	Participant interviews, observational records
	Experiences of fitting and misfitting and the boundaries of participation	Participant interviews, observational records
	Public recognition and the internalization of responsibility	Participant interviews, caregiver interviews
From performance to everyday life: continuity of performance experience	Reorganization of self-evaluation	Participant interviews, observational records
	Extending self-expression Into everyday life	Participant interviews, caregiver interviews, and observational records
	Conditions supporting and constraining continuity	Participant interviews, caregiver interviews, and observational records

### 4.1 Embodied agency formation

This theme concerns how repeated rehearsal, instructor feedback, and coordination with other dancers shaped participants' growing familiarity with movement and shared performance.

#### 4.1.1 Repeated performance and the reconstruction of possibility

Repeated performances allowed participants to gradually become familiar with their bodies and the dance space. Movement and performance were not experienced as familiar from the outset. Rather, through repeated attempts, participants gradually developed a practical familiarity with how they could move and perform.

Doyoon described a shift from unfamiliarity and fear to familiarity within the dance space:

“*At first, the big mirror felt unfamiliar and scary, but now I'm used to it, and I can dance like a swan*.”

Initially, the large mirror and rehearsal space were unfamiliar and intimidating to Doyoon. Through repeated rehearsal, however, he became accustomed to the environment and increasingly comfortable performing within it, reflecting a gradual shift from hesitation to familiarity.

Yujin, too, framed repeated performance as the process of trying again while regulating tension and embarrassment:

“*When they said, ‘Let's try again,' I worked really hard… The teacher told me to relax and do it, and then I tried hard…*”

Rather than stopping after a mistake, Yujin attempted the movement again following the instructor's encouragement. Repetition therefore involved continuing to participate despite tension, embarrassment, and uncertainty.

Repeated performance was also evident in our observations, where physical discomfort became an occasion to return to practice rather than to stop.

“*During the performance, Yujin had a cramp in her calf… She sat down on the spot but soon stood up again and participated in dance practice… The instructor told her, ‘Go to the side and rest for a bit,' but she refused and returned to practice*.”

Although offered the opportunity to rest, Yujin returned to rehearsal after the temporary interruption and continued practicing. Her return showed that physical discomfort could interrupt participation without necessarily bringing the rehearsal experience to an end.

Caregiver accounts also showed repeated performance as sustained concentration and maintenance of performance rules:

“*Jia really works the hardest… In the dance studio, whenever they decide the order, she is always first… When the movements change, others sometimes cannot remember them… But Jia remembers all the changes… At the end, everyone else stood up, but Jia was the only one who stayed seated and bowed*.”

Jia's sustained attention to movement sequences and rehearsal routines was also evident in her caregiver's account. Through continued participation, she consistently remembered changes in choreography and maintained the expected order of performance, even when other dancers had difficulty recalling the changes.

Across interviews, observations, and caregiver accounts, repeated performance took several forms: becoming familiar with the rehearsal environment, returning after interruption, and maintaining changing movement sequences and performance routines. These patterns indicate that familiarity with dance practice developed gradually through continued participation despite unfamiliarity, tension, physical interruption, and changes in choreography.

#### 4.1.2 Feedback and the embodiment of performance rules

Feedback guided how the participants regulated their bodies and responded within the performance settings. Instructors' explanations, repeated requests, movement corrections, and encouragement helped participants understand movement sequences and performance rules and adjust their own bodies accordingly.

Doyoon recounted how the instructors' explanations and repeated practice shaped his participation:

“*When they taught me the movements a lot… they played the music, right? I think they did that so we could memorize it… As the teachers came from far away, I thought I should work hard too… The teacher explains, right? When the others cannot focus, I take them one by one and tell them to go forward*.”

Beyond following the instructor's explanations himself, Doyoon took on a guiding role toward others, reminding fellow dancers of their positions when they lost focus. Feedback thus extended beyond the regulation of his own performance as he drew on the instructor's directions when helping other dancers participate in the rehearsal.

Jia experienced the instructor's feedback as a process of learning the details of the movement:

“*The teacher was kind… The teacher was good to me… She taught me well in detail*.”

For Jia, feedback was experienced through the instructor's detailed and supportive guidance. Her emphasis on being taught “well” and “in detail” placed the manner of instruction alongside the movement guidance itself as part of her rehearsal experience.

The internalization of feedback was also identified in the observation data:

“*Even when sitting down, when the instructor called Doyoon's name, he quickly stood up and answered, ‘Yes.' When the instructor showed a dance movement, he immediately responded, ‘Yes, I understand. I'll try it once.'*”

The observational data further demonstrated how Doyoon responded to such guidance during rehearsal. When his name was called or a movement was demonstrated, he responded promptly and attempted the movement as instructed, showing a consistent readiness to follow the instructor's cues.

Feedback operated through repeated explanation, correction, demonstration, and encouragement, supporting participants' growing familiarity with movement sequences and rehearsal procedures as well as their practical responses to the expectations that organized performance.

#### 4.1.3 Relational coordination and the confirmation of agency

Agency was evident in relational moments when the participants moved together, attended to one another's condition, and coordinated with each other's movements and emotions. For the participants, rehearsal and performance involved attending to others, responding to peers, and continuing activities together within the shared dance setting.

Jia described dance participation as being with peers who shared similar experiences:

“*In the dance company, I can do it together with friends with disabilities… If we don't do it all together, I feel lonely*.”

For Jia, participation was closely tied to being with other dancers. Her loneliness when the group did not dance together marked dancing as a shared activity rather than an individual one.

Doyoon, likewise, cast agency as the practice of caring for and coordinating with others:

“*I want to take good care of them… I thought I should help them. While performing together, we also talk with each other*.”

For Doyoon, coordinating with others involved helping, talking, and remaining attentive to fellow dancers while performing together. He described this relational dimension of dance more explicitly as a form of bodily communication:

“*Dance is like speaking. It is speaking with the body with friends who dance together*.”

By describing dance as “speaking with the body,” Doyoon presented shared performance as a way of communicating with others through movement. His account connected participation not only with performing his own movements but also with maintaining interaction with fellow dancers.

Yujin, too, pointed to care and bodily interaction as important relational experiences in dance:

“*A friend I do activities with came to me and asked me to hug them… Like the teachers, I rubbed my friends' feet when they had cramps*.”

Yujin's account added a more practical form of relational coordination. Hugging a friend and rubbing a peer's feet during physical discomfort showed how care for others was incorporated into the everyday interactions surrounding rehearsal.

Relational coordination was also identified in the observational data:

“*One dancer disliked loud sounds. When there was a sudden loud noise, the dancer started to cry. Jia approached and calmly comforted the dancer, saying, ‘It's not because they dislike you,' and ‘The teacher is not angry.' She helped the dancer return to performance practice*.”

This coordination was also visible in the observational data. When another dancer became distressed and temporarily withdrew, Jia approached, reassured the dancer, and remained engaged until the dancer returned to practice. This episode extended relational coordination beyond shared movement to include responding to a peer's emotional condition in ways that supported continued participation.

Across the interviews and observation, relational coordination took the form of dancing together, communicating through movement, attending to peers' physical and emotional conditions, and helping others remain involved in rehearsal. These experiences show that participation within the dance setting was sustained not only through individual performance but also through ongoing responsiveness to others.

### 4.2 Normative visibility and conditions of participation

This theme addresses how roles, titles, public recognition, and opportunities for participation shaped participants' positions within rehearsal and performance settings.

#### 4.2.1 Legitimizing participation through naming and role allocation

In inclusive dance settings, participation involved more than being physically present in rehearsal or performance spaces. Participants described how names and roles such as “dancer,” “assistant teacher,” and “teacher” distinguished their positions and clarified what they were expected to do within the group. Being cast or addressed through a particular role also allowed them to experience themselves as people who could perform, teach, or assist others.

Jia recounted how her participation on stage became meaningful through role allocation:

“*The professor cast me… I felt good… I wanted to be an instructor… because I could teach the children who could not do it well*.”

Being “cast” gave Jia a defined place in the performance rather than simply allowing her to attend the activity. The role also extended how she positioned herself within the dance setting, from someone receiving instruction to someone who could teach and support children who experienced difficulty.

For Doyoon, naming was directly connected to an experience of recognition:

“*When they call me teacher, I feel good… It feels like I'm being recognized by the teacher? … The children's mothers don't call me just Doyoon. They call me Teacher Doyoon, so I liked that. It felt like being recognized*.”

For Doyoon, the form of address itself was central to the experience of recognition. Being called “Teacher Doyoon” rather than simply by his name signaled that others regarded him as someone who could teach and assist within the dance setting.

Role allocation was also connected to experiences of burden. Yujin spoke of the assistant teacher's role in more ambivalent terms:

“*I didn't want to be an assistant teacher… I felt burdened about becoming a teacher… I worried I might not teach well… I'm not good either, but I had to take care of the children*.”

Yujin experienced the designation of “assistant teacher” more ambivalently. The role brought worries about teaching adequately and caring for the children, showing that an assigned position could carry responsibilities alongside the recognition associated with it.

These roles were also observed in actual performance settings:

“*The participant went around checking other dancers' condition before practice… During dance performance practice, the participant provided feedback to a young dancer appearing in the same scene, such as ‘Come closer' and ‘Well done'*.”

The observational data showed how these assigned roles were enacted in practice. The participant checked other dancers' conditions before rehearsal and guided a younger dancer through positional cues and encouragement, demonstrating that role allocation was expressed through concrete acts of attending to and supporting others.

Considered together, the interviews and observational data indicate that naming and role allocation distinguished participants' positions within the dance setting and were accompanied by specific expectations. Being cast or called “teacher” provided experiences of recognition and expanded participants' involvement beyond performing their own movements. At the same time, roles such as “assistant teacher” brought concerns about teaching accurately, caring for others, and fulfilling the responsibilities attached to the role.

#### 4.2.2 Experiences of fitting and misfitting and the boundaries of participation

Participants encountered boundaries to participation through unequal stage opportunities, comparison with non-disabled dancers, and interactions during rehearsal. These boundaries were experienced in relation to performance opportunities, expectations, instructional approaches, and the organization of the performance setting.

Jia recalled how opportunities to participate on the stage were restricted in a previous dance environment:

“*Only the other major-class students went to competitions. The director did not include me… Only the non-disabled friends kept going to competitions… So I wondered whether I was not good enough. Until then, I thought it was embarrassing*.”

For Jia, repeated exclusion from competition led her to question her own ability. Although the opportunity itself was determined within the dance environment, she initially experienced that exclusion through self-doubt and embarrassment.

A similar pattern appeared in Doyoon's experience:

“*Other friends stood on the competition stage, wore pretty costumes, and performed, but I could not wear a pretty costume or stand in the competition. I felt a little despair*.”

For Doyoon, exclusion was experienced through concrete features of performance participation—the competition stage, performance costumes, and the opportunity to perform alongside other dancers. His sense of despair was therefore connected not only to being excluded from competition but also to being unable to take part in these visible aspects of performance.

Yujin's account shows how the experience of boundary-making did not disappear completely in the current participation setting:

“*Because people with disabilities dance together… When people with disabilities stand with non-disabled people, they feel embarrassed. It's not just me*.”

Yujin's account located embarrassment in situations where dancers with disabilities performed alongside non-disabled dancers. By adding that this feeling was “not just” hers, she described comparison with non-disabled dancers as an experience that extended beyond her individual response.

The mode of interaction in performance settings also appeared to be a boundary condition of participation.

“*During performance practice… the instructor pushed one dancer as if scolding them… The dancer disliked loud sounds, so the dancer suddenly began to cry*.”

Observation revealed a different form of boundary during rehearsal. A forceful correction in a situation involving a dancer who was sensitive to loud sounds caused visible distress and temporarily interrupted that dancer's participation. This episode showed that participation could also be affected by how instruction and interaction were organized within the rehearsal setting.

Across the data, boundaries to participation appeared through unequal access to competition and performance resources, comparison with non-disabled dancers and forms of interaction during rehearsal. These different conditions were associated with self-doubt, embarrassment, despair, and temporary interruption of participation.

#### 4.2.3 Public recognition and the internalization of responsibility

Participants described how public recognition and assigned roles were accompanied by expectations about how they should perform. Praise from others and responsibilities associated with particular roles were often experienced together during rehearsal and performance.

Jia's account reflects the internalization of this responsibility:

“*I feel burdened when the children look at me… If I am an assistant teacher, I shouldn't make mistakes… If I make a mistake, the children might get confused*.”

For Jia, being watched as an assistant teacher brought a responsibility to perform without mistakes because she was concerned that her mistakes might confuse the children she supported. The attention she received was therefore accompanied by an increased awareness of how her own performance could affect others.

For Doyoon, public recognition extended into responsibility for the group:

“*If I don't do it, the children won't do it… A volunteer told me… if Teacher Doyoon is not there, the children won't do it*.”

Doyoon encountered a different form of responsibility. Being told that the children might not dance in his absence indicated that his participation had become important to their continued involvement. Recognition of his role was thus accompanied by an expectation that he would remain present and support the participation of others.

Caregiver accounts similarly suggested experiences of responsibility and burden.

“*When people say, ‘You're good, you're good,' Doyoon tries to do even better… Now he seems to feel more responsibility… He seems to feel a lot of responsibility*.”

Doyoon's caregiver observed that positive evaluations were followed by greater effort and a stronger sense of responsibility. From the caregiver's perspective, praise was therefore associated not only with affirmation but also with Doyoon's increasing concern about performing his role well.

Across participant and caregiver accounts, recognition did not remain simply a positive evaluation. Being watched, praised, or regarded as important to others was accompanied by expectations to perform well, remain involved, and support other dancers. Responsibility therefore emerged alongside recognition as participants became increasingly aware that their own performance and presence could affect others within the dance setting.

### 4.3 From performance to everyday life: continuity of performance experience

The third theme concerns how performance experiences extended beyond the stage into self-evaluation, everyday expression, and the practical conditions of continued participation.

#### 4.3.1 Reorganization of self-evaluation

Performance experiences provided opportunities for participants to reflect on their own performances. Audience responses, instructors' feedback, and performance experiences were frequently mentioned when participants described how they felt about their performances after appearing on stage.

Yujin's account directly reflects a reorganization of self-evaluation:

“*When they said I did well, I felt like I really had done well. And when many people in the audience clapped, I felt really good*.”

Yujin's positive self-evaluation was closely connected to others' responses to her performance. Being told that she had performed well and receiving audience applause reinforced her own sense that she had done well.

For Doyoon, self-evaluation appeared through performance standards and expectations:

“*I usually don't make mistakes, so I wanted to receive a lot of applause*.”

Doyoon connected self-evaluation with both his own expectation of performing without mistakes and the audience response he hoped to receive. Unlike Yujin, whose account emphasized others' positive responses, Doyoon referred to a performance standard he already applied to himself alongside his expectation of applause.

A similar pattern appeared in Jia's account:

“*First of all, I think it would be good if people paid attention to me… It makes me feel more confident. I think I would feel really good if I heard, ‘Jia, you're doing well*.”'

Jia associated being noticed and positively evaluated by others with greater confidence in her performance. Her account emphasized attention and verbal affirmation as important to how she felt about herself as a performer.

Changes in self-evaluation were also noted in the observational data:

“*Jia arrived earlier than the practice time and was excited, saying that she had a solo in today's performance practice. She went around telling the other dancers, as if proudly showing it off*.”

The observation added another dimension to Jia's self-evaluation. Her excitement about having a solo and her repeated announcement of it to other dancers provided an observable expression of the pride she associated with being given a distinct performance opportunity.

Self-evaluation took several forms across participants: satisfaction following applause and praise, confidence associated with positive attention, expectations about performing without mistakes, and pride in being given a solo role. Overall, participants evaluated their performances through both their own expectations and the responses and opportunities they encountered within the performance setting.

#### 4.3.2 Extending self-expression into everyday life

Participants described how their dance experiences continued beyond rehearsals and performances into everyday conversations, clothing, and interactions with others. They talked about dance not only during performances but also when describing themselves and sharing their experiences in daily life.

Doyoon explained that he wanted more people to know about his dance experience:

“*I want to let the world know… I want to let more people know*.”

Doyoon's repeated wish to “let more people know” conveyed a desire to share dance beyond the rehearsal and performance setting. Rather than keeping the experience private, he expressed a desire to make his participation known to others.

For Jia, self-expression appeared more concretely in her awareness of performance costumes.

“*Because I can wear pretty costumes… Are pretty clothes and pretty costumes different? Yes… Clothes are what you wear in everyday life. But costumes are what you wear only for performances. So I realized they are different*.”

Jia clearly distinguished clothes worn in everyday life from costumes worn specifically for performance. For her, performance attire had taken on a distinct meaning within her dance experience, marking the stage as different from ordinary daily settings.

The expansion of self-expression was also evident in the caregivers' accounts. Doyoon's caregiver explained that he often talked about his dance activities in everyday conversations:

“*He often speaks proudly about the fact that he dances now… He talks about where he dances and things like that… He even talked about it at work, and once they asked him to send photos, so we sent some*.”

The caregiver's account provided a concrete example of dance extending into Doyoon's everyday interactions. He spoke about dancing in different settings, including at work, and shared photographs of his performances when others showed interest.

The observational data also showed the expansion of self-expression in participants' everyday preparation for performance.

“*Other dancers practiced in the clothes they had worn when coming to the dance company, but Yujin was different because she changed into dancewear that she had brought separately before beginning*.”

Yujin's preparation for rehearsal provided another form of this extension. By bringing separate dancewear and changing before practice, she incorporated dance into the routines and preparations surrounding rehearsal, rather than limiting it to the performance itself.

Dance extended beyond the stage through talking about participation, sharing performance photographs, distinguishing performance attire from everyday clothing, and preparing specifically for rehearsal. In these various ways, dance became present in participants' everyday interactions and routines.

#### 4.3.3 Conditions supporting and constraining continuity

Participants described how continuing dance participation depended on a range of everyday conditions, including their physical condition, daily schedules, transportation, family support, and rehearsal arrangements. Although they expressed a desire to continue dancing, their participation was influenced by these practical conditions.

Jia explained that her body's condition was an important factor in her dance participation:

“*What was difficult was… it was a bit hard when I had a cold… and when I was coughing a lot, I said I was a little sick…*”

For Jia, continuity was contingent on her physical condition. Illness and persistent coughing made participation more difficult and could temporarily limit her involvement in dance activities.

Yujin pointed to time as a constraint on continuity:

“*I wish the class duration were a little shorter because I have many things to do. Sometimes I feel that the dancing session has become a little long*.”

For Yujin, continued participation had to be accommodated within other daily commitments. Her concern about class duration pointed to how the length and timing of dance sessions could affect how easily participation fitted into everyday life.

Continued participation was also connected to family support. Her caregiver stated:

“*Because her mother said it was hard, she thought she should stop… When I go to pick her up, I also come after work… Honestly, it is hard*.”

The caregiver's account located part of that continuity outside the studio. Transportation and assistance after work enabled participation but also placed demands on the family members providing that support.

The observational data also showed a desire to continue by remaining connected to the setting while adjusting body conditions:

“*Although the participant was observed sitting and resting from time to time due to physical fatigue, the participant's gaze remained directed toward the practice room. This suggested an attitude of adjusting bodily condition and returning to practice rather than completely withdrawing from participation*.”

The observational data added another dimension to continuity. The participant temporarily rested because of fatigue but remained oriented toward the rehearsal and returned when able to continue, showing that sustained participation could include periods of adjustment rather than uninterrupted involvement.

Taken together, the interviews, caregiver accounts, and observations presented continuity as something negotiated through bodily condition, time, transportation, and family support. Participation therefore varied with everyday circumstances, even when participants remained willing to continue attending rehearsals and performances.

## 5 Discussion

Our study suggests that agency, visibility, identity, and wellbeing were not separate dimensions of inclusive dance participation among adults with developmental disabilities but were co-constituted within this participatory experience. Through inclusive dance as a practical setting, the participants we interviewed experienced the possibilities of their bodies and reevaluated themselves within these public performances and in their everyday relationships. This interpretation positions inclusive dance as an experiential process in which embodied agency, relational self-understanding, and conditions for sustained participation intermingle.

### 5.1 Organizing embodied agency: the relational reconstruction of “I can”

The sense of “I can” in inclusive dance is best understood not as an individual capacity but as a relational accomplishment emerging through ongoing interactions between bodies, others, and the participation environment. This was evident in participants' gradual movement from an initial unfamiliarity with the rehearsal space toward a felt capacity to act within it. Embodied agency developed through sustained participation within relational and environmental conditions, aligning with phenomenological and disability-phenomenological perspectives that conceptualize agency as an embodied process constituted through engagement with the world rather than residing solely within the individual (20, 26, 27). Accordingly, Merleau-Ponty's notion of “I can” can be understood as extending beyond bodily competence to encompass the continual negotiation of participation within shared performance contexts. This reading is consonant with disability-studies work that reframes what a body can do as arising within material and relational conditions rather than as an attribute of the individual, moving beyond narratives of individual deficit or overcoming (13).

Repeated participation therefore represents more than the refinement of technical movement. Phenomenological discussions of body schema and habit suggest that embodied familiarity develops gradually through repeated engagement with meaningful practices (20). In our findings, this gradual reorganization appeared as participants grew familiar with rehearsal routines and returned to practice following interruptions, so that movements once experienced as unfamiliar became available as possibilities for action. Our findings contribute to this perspective by indicating that inclusive dance provides opportunities for embodied possibilities to emerge through sustained participation in relational performance environments rather than through movement practice alone. This interpretation also extends previous discussions of dance as embodied learning and self-understanding by emphasizing the relational organization through which bodily possibilities are gradually constructed (21, 22).

A further implication concerns the relationship between vulnerability and agency. Recent disability phenomenology has increasingly challenged the assumption that agency depends upon bodily autonomy or the absence of vulnerability (26, 31, 48). Instead, vulnerability is understood as an ordinary condition of embodied existence through which new forms of participation become possible. In our findings, participants sustained their engagement through moments of tension, hesitation, and bodily discomfort rather than withdrawing from participation. Our findings support this perspective by suggesting that embodied agency was constituted through the ongoing negotiation of bodily uncertainty within supportive participation environments rather than through the elimination of vulnerability itself.

Contemporary phenomenological accounts likewise emphasize that agency emerges through participatory coordination and interdependent relationships rather than through isolated individual action (27, 49). This was apparent in participants' mutual attentiveness during rehearsal, as they coordinated their movements, participated together, and responded to one another's emotional and physical needs. Viewed in this way, embodied agency is collectively organized through reciprocal adjustment, shared practices, and relational forms of care that sustain participation over time. Inclusive dance thus illustrates how bodily capability is continually constituted through interactions among bodies, relationships, and environments.

Overall, these findings shift attention from the outcomes of participation to the relational conditions through which embodied agency becomes possible. This interpretation contributes to arts and health research by demonstrating that bodily possibility is organized through sustained interactions among bodies, environments, and social relationships rather than through individual competence alone. As the foundation of participation, embodied agency also provides the basis upon which subsequent processes of public recognition, everyday self-understanding, and wellbeing become possible within inclusive dance.

### 5.2 Normative visibility, public recognition, and role-based self-understanding

Normative visibility concerns the social processes through which particular bodies become recognizable, legitimate, and meaningful within specific social contexts rather than the simple fact of being publicly seen (50). Viewed in this way, recognition is not an individual achievement but a relational accomplishment shaped by the normative organization of social practices, material environments, and institutional expectations. Relational perspectives on disability similarly emphasize that bodily difference is constituted through interactions between bodies and their social and material environments rather than through impairment itself (14, 24). Contemporary disability embodiment scholarship further suggests that bodily capability emerges through relationships, everyday practices, and networks of care rather than residing solely within autonomous individuals (25–27). Collectively, these perspectives shift attention from individual bodily characteristics to the relational conditions through which participation becomes socially intelligible.

These conditions were not only social but also material and spatial. In our findings, the boundaries of participation formed around access to competition stages, performance costumes, and placement alongside or apart from non-disabled dancers—material arrangements that made some forms of participation available while limiting others. Garland-Thomson's concept of misfitting is instructive here, since it situates disability in the encounter between a body and a material environment that either sustains it or fails to, rather than within the body alone (14). From this perspective, exclusion followed less from participants' capacities than from settings in which costumes, staging, and spatial arrangements were organized around bodily expectations. Ahmed's notion of orientation extends this reading: spaces, objects, and established pathways direct bodies, rendering some movements and forms of presence familiar and readily accommodated while positioning others as out of place (24). Together, misfitting and orientation recast normative visibility as a materially and spatially organized process, in which a body's recognizability as a legitimate performer depends on how the material conditions of performance are arrange to receive it.

Recognition can therefore be understood not as the endpoint of inclusion but as an ongoing social process through which participation acquires legitimacy and meaning. Becoming publicly visible also involves becoming situated within normative expectations regarding responsibility, contribution, and social participation (30, 31). In our findings, this double character was evident in how naming and the allocation of roles conferred recognition while, at the same time, placing expectations and a felt responsibility toward others on participants. In this sense, public recognition is not merely external acknowledgment but also a process through which individuals come to understand themselves in relation to others. Role-based self-understanding therefore emerges as participants interpret themselves through socially recognized roles and forms of participation. Recognition thus shapes both public visibility and how individuals understand their place, responsibilities, and possibilities within shared social worlds.

Our findings contribute to these discussions by suggesting that inclusive dance should be understood not simply as a context in which disabled bodies become visible but as a practice that reorganizes the relational and material conditions through which recognition itself becomes possible. Visibility therefore emerges neither as an individual attribute nor as a direct consequence of bodily performance. Rather, it is constituted through the continuing interaction of bodies, environments, and social practices that collectively shape how participation is recognized and valued. Within these relational processes, public recognition also provides an interpretive framework through which participants reconstruct their own roles and identities as socially recognized contributors rather than passive recipients of support. This interpretation extends disability embodiment and arts-based health research by demonstrating that recognition simultaneously reorganizes public visibility and role-based self-understanding through shared participation.

Overall, these findings indicate that public recognition is best understood as a relational process through which embodied participation becomes socially meaningful. Inclusion in this context is therefore sustained not because bodily difference disappears but because the conditions governing recognition are continually reorganized to support meaningful participation. In this respect, our interpretation resonates with critiques of inclusionism, which caution that inclusive initiatives may admit some disabled people—often those who most readily perform competence or meet prevailing expectations—while leaving the structural terms of participation unchanged for others (51). Understood in this light, the experiences examined here describe the relational and material conditions under which participation became possible for these particular participants, rather than demonstrating that inclusion has been secured. Recognition also provides the relational foundation through which participants reinterpret themselves as legitimate social actors, allowing embodied experience to continue beyond performance and into everyday self-understanding.

### 5.3 Everyday reconstruction of performance experience: from the stage to the everyday world

Performance should not be understood as a discrete event that concludes when a performance ends. Rather, performance experience can be interpreted as an ongoing process through which embodied meaning is reconstructed within everyday life. From a phenomenological perspective, embodied experience is never confined to a particular moment but continues as individuals reinterpret themselves through their ongoing engagement with the world (20). This perspective shifts attention from the performance itself to the ways in which its meaning is continually reconstituted through everyday relationships and practices.

This interpretation also extends current discussions in dance and health research, where the value of dance participation has largely been explained through improvements in physical function, emotional wellbeing, social connectedness, and quality of life (1, 52, 53). While these studies have established the beneficial outcomes of dance participation, less attention has been given to how performance experience itself continues beyond the immediate context of participation. In our findings, participants reappraised their own performances through applause and encouraging feedback from audiences and instructors, which gradually informed how they understood themselves. A phenomenological perspective suggests that embodied experience remains meaningful because it is continually reinterpreted within subsequent social encounters and everyday practices.

From this perspective, everyday self-understanding is not simply a psychological reflection on past experience but an ongoing embodied process through which individuals negotiate their relationships with others. In our findings, this was visible as participants spoke about their dancing with those around them and drew a distinction, in their own self-presentation, between the dress of the stage and that of everyday life. Previous work in disability arts has similarly emphasized that artistic participation can transform how disabled bodies are expressed, interpreted, and recognized within everyday social life (54–56). Rather than treating performance as an isolated artistic achievement, these discussions locate its significance within the continuing reconstruction of social identity and embodied participation.

Our findings extend these discussions by suggesting that the significance of inclusive dance lies not only in participation or public performance but also in the continued reinterpretation of embodied experience within everyday life. In our findings, participants carried performance memories and their own accounts of dancing into everyday interactions, so that performing remained a meaningful reference through which bodily possibility, self-expression, and social relationships were continually reconfigured. This interpretation broadens existing discussions of dance and wellbeing by locating wellbeing within sustained processes of embodied meaning-making rather than within the immediate outcomes of participation alone.

Taken together, these findings suggest that performance experience is best understood as an ongoing process of everyday reconstruction rather than a completed event. The significance of inclusive dance therefore lies in the continuity of embodied self-understanding, through which the meaning of performance is sustained beyond the stage and integrated into everyday life.

### 5.4 Conditions of wellbeing: sustaining participation with vulnerable bodies

Wellbeing should not be understood solely as an individual psychological outcome of arts participation but as a relational and environmental condition that enables sustained participation. From this perspective, wellbeing emerges through the social, material, and temporal conditions that allow individuals to remain engaged despite changing bodily circumstances. This shifts the focus from what participation produces to the conditions that make participation itself possible. In our findings, continued participation depended on such conditions: fluctuations in health and energy, the demands of daily schedules, and the transportation and family support required to keep attending. This interpretation extends current discussions in the arts and health literature, where wellbeing has primarily been examined in relation to emotional stability, social connectedness, quality of life, and physical functioning (1, 52). Although these studies have established the value of arts participation for health, they have paid less attention to the conditions through which participation can be sustained over time. A relational understanding of wellbeing suggests that health-related benefits are inseparable from the environments and social relationships that enable continued participation.

This perspective is consistent with recent developments in disability studies and inclusive health research, which increasingly conceptualize wellbeing as emerging through accessibility, interdependence, relational support, and environmental responsiveness rather than through individual functioning alone (26, 27, 31, 57). Within this framework, care is understood not as an additional form of support but as a constitutive condition of participation itself (28). Likewise, sustainability depends upon the capacity of participation settings to accommodate bodily variability through flexible social and material arrangements rather than through expectations of bodily consistency or independence (58).

The present study contributes to these discussions by suggesting that inclusive dance provides an example of how wellbeing is constituted through the ongoing organization of relationships, care, and participation rather than through the achievement of predetermined health outcomes. This perspective moves beyond conventional outcome-oriented approaches by conceptualizing wellbeing as a condition that enables participation to continue despite vulnerability and bodily change. In our findings, this was visible as participants managed fatigue and discomfort by pausing and rejoining rather than withdrawing, and were supported at home and within the group in ways that made returning possible. Accordingly, participation is sustained not because vulnerability is overcome but because the participation environment is continually reorganized in response to changing bodily circumstances.

Overall, the present study suggests that inclusive dance supports wellbeing not simply by improving individual outcomes but by organizing the relational conditions through which embodied agency, public recognition, continuity of self-understanding, and sustained participation become possible. This perspective contributes to disability embodiment and arts-based health research by conceptualizing wellbeing as the relational achievement of environments that enable diverse bodies to participate, belong, and continue over time.

## 6 Conclusions

We analyzed the inclusive dance experiences of adults with developmental disabilities using embodied agency and critical phenomenology as our theoretical foundations. Our findings suggest that inclusive dance is a process through which participants experience their bodies as capable of performing and re-envision themselves through relational coordination and public recognition.

The meaning of inclusive dance extends beyond participation in artistic activities or performance outcomes. Through repeated performance, role experience, interactions with others, and everyday self-expression after performances, participants reconstructed their bodily possibilities and social presence in everyday life. Simultaneously, these experiences were sustained through bodily conditions, care relationships, temporal structures, mobility support, and environmental flexibility.

Our study suggests that inclusive dance should be understood as a participatory experience in which the body, relationships, norms, and everyday world intersect for adults with developmental disabilities. The study's contribution lies in interpreting the value of arts participation as a process in which embodied agency, normative visibility, relational self-understanding, and conditions for sustained participation are closely interconnected, rather than only as intrapersonal psychological changes or program effects. This perspective expands current discussions of arts participation and wellbeing by emphasizing the relational and environmental conditions through which participation becomes sustainable in everyday life.

The findings should be interpreted within the specific context of this study, which involved participants from a single inclusive dance organization. In addition, all participants had extensive experience in inclusive dance, and the relatively homogeneous sample and purposive recruitment may have introduced selection bias. Participants' responses may also have been influenced by socially desirable reporting. Future research may examine how embodied agency, recognition, and wellbeing are experienced across different inclusive arts settings, disability groups, and cultural contexts.

## Data Availability

The data presented in this study are available on request from the corresponding author. Some information cannot be shared to preserve the anonymity of the participants. Requests to access the datasets should be directed to HA, ahnhk@mjc.ac.kr.
